# A systematic review of quality of life instruments in long-term breast cancer survivors

**DOI:** 10.1186/1477-7525-10-14

**Published:** 2012-01-31

**Authors:** Ishveen Chopra, Khalid M Kamal

**Affiliations:** 1 Division of Clinical, Social and Administrative Sciences, Mylan School of Pharmacy, 418F Mellon Hall, Duquesne University, 600 Forbes Avenue, Pittsburgh, PA 15282, USA; 2 Division of Clinical, Social and Administrative Sciences, Mylan School of Pharmacy, 418B Mellon Hall, Duquesne University, 600 Forbes Avenue, Pittsburgh, PA 15282, USA

**Keywords:** Breast cancer, breast cancer survivors, quality of life, instruments

## Abstract

**Background:**

Breast cancer is the most common cancer in women, representing 16% of all female cancers. According to the American Cancer Society, long-term cancer survival is defined as more than five years of survivorship since diagnosis, with approximately 2.5 million breast cancer survivors (BCS) in 2006. The long-term effects from breast cancer and its treatment have been shown to have positive and negative effects on both recovery and survivors' quality of life (QoL). The purpose of the study was to identify QoL instruments that have been validated in long-term BCS and to review the studies that have used the QoL instruments in this population.

**Methods:**

A systematic literature search was conducted from January 1990 to October 2010 using electronic databases. Instruments validated and used in BCS were included in the review. In addition, QoL studies in long-term BCS using the validated instruments were reviewed. The search was limited to studies in English language. Studies of BCS of less than five years after initial diagnosis, any clinical or review studies were excluded.

**Results:**

The review identified a total of 12 instruments (10 disease-specific, 2 condition-specific) validated in long-term BCS. According to the QoL framework proposed by Ferrell and colleagues, three instruments (Quality of Life-Cancer Survivors, Quality of Life in Adult Cancer Survivors Scale, and Quality of Life Index-Cancer Version) evaluated all four domains (physical, psychological, social, and spiritual) of QoL. A review of the psychometric evaluation showed that Quality of Life in Adult Cancer Survivors Scale has acceptable reliability, validity, and responsiveness in long-term BCS compared to other disease-specific instruments. The review also yielded 19 studies that used these QoL instruments. The study results indicated that age-group, ethnicity, and type of treatment influenced different aspects of QoL.

**Conclusions:**

There is a significant impact of breast cancer on QoL in long-term BCS. The review can help researchers and clinicians select the most appropriate instruments to assess the changes in QoL in BCS.

## Introduction

Breast cancer is the most common cancer in women, representing 16% of all female cancers [1]. Approximately 200,000 new cases of breast cancer are diagnosed each year in the United States (US) [2]. Most significant risk factors for the disease include age, gender, and race/ethnicity [3]. Breast cancer incidence and death rates generally increase with age; women older than 45 years are at the greatest risk [3]. In developed countries, there has been a significant decline in the mortality rate due to improved diagnosis and treatment programs. The National Cancer Institute estimated approximately 2.5 million breast cancer survivors (BCS) in the US in 2006 [4].

The long-term effects from breast cancer and its treatment have been shown to have positive and negative effects on both recovery and survivors' quality of life (QoL). Also, QoL outcomes vary across the breast cancer continuum including diagnosis at different stages of breast cancer, disease-free survivorship beyond the first course of primary treatment, long-term disease-free survivorship, and first recurrence of breast cancer [5]. According to the American Cancer Society (ACS), a long-term cancer survivor is defined as an individual who has survived five or more years since the diagnosis of cancer [6]. Long-term difficulties resulting from breast cancer differ from those experienced during diagnosis and treatment. Breast cancer patients are at an increased risk of developing physical conditions (*e.g*., fatigue, sleep disturbances, and pain) and psychological distress (*e.g*., depression, anxiety, negative thoughts, fear of cancer recurrence and death, sense of aloneness, sexual, and body image problems) after diagnoses that adversely affect their overall QoL and survivorship [7].

The implementation and use of improved diagnosis and treatment programs have resulted in decreased breast cancer mortality [8]. However, these new long-term therapies have persistent unknown side-effects and toxicity, which have negatively impacted survivor's QoL [8]. Different therapies including surgery, systemic therapies (chemotherapy, hormone therapy, radiation therapy, and newer targeted therapies with monoclonal antibodies), and adjuvant endocrine therapy have varied QoL outcomes [8]. Breast cancer surgery is associated with lasting effects including pain, fatigue, and psychosocial distress. The treatments involve the use of more toxic and multimodal regimens with little focus on long-term effects of therapies. Fatigue, weight gain, lymphedema, pain, and menopausal symptoms are long-term effects that result from systemic therapies. The use of anthracyclines and adjuvant trastuzumab have been linked to the risk of developing cardiac problems even after the treatment has ended, whereas women on aromatase inhibitors are at an increased risk of bone loss and fractures [9]. The radiation therapy is linked to the potential development of sarcomas [9]. It has been reported that lack of knowledge in recovery patterns and evidence-based guidelines for follow-up care mostly result in persistent and late effects of cancer treatment [7].

**Figure 1:**
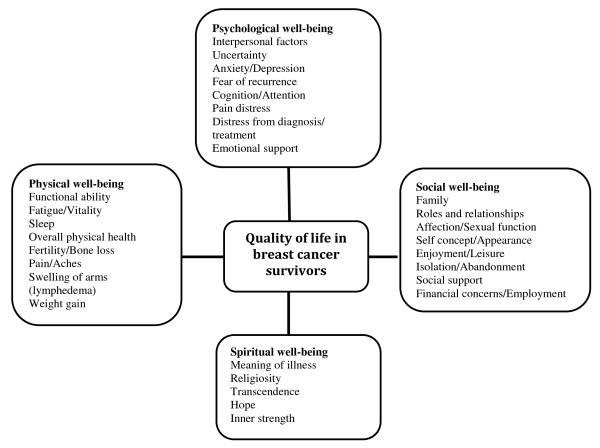
**Specific quality of life model for breast cancer survivors**. Adapted with permission from Betty Ferrell & Marcia Grant: Quality of Life Conceptual Model Applied to Cancer Survivors, City of Hope Beckman Research Institute [10].

The problems resulting from breast cancer and its treatment are varied and complex. Ferrell *et al *[10] proposed a QoL model for long-term cancer survivors [7] (Figure 1) that comprises of four primary domains of well-being (psychological, social, physical, and spiritual) that are integrally related and relevant to BCS. Thus, for a better understanding of the long-term impact of cancer diagnosis and its treatment, it is important to examine all the four domains of a survivor's well-being. In addition, the health perceptions and expectations have been found to vary with person's age, experiences, gender, and health history [11]. This further necessitates the recognition of specific medical and psychosocial needs so as to optimize health promotion in survivors.

### Objectives

The specific objectives of this review were: (1) to identify QoL instruments validated and used in long-term BCS; (2) to provide a description of the instruments and their psychometric properties (reliability, validity, and responsiveness) in long-term BCS; and (3) to provide a systematic review of studies that used the QoL instruments in long-term BCS.

## Methods

### Search strategy

**Figure 2:**
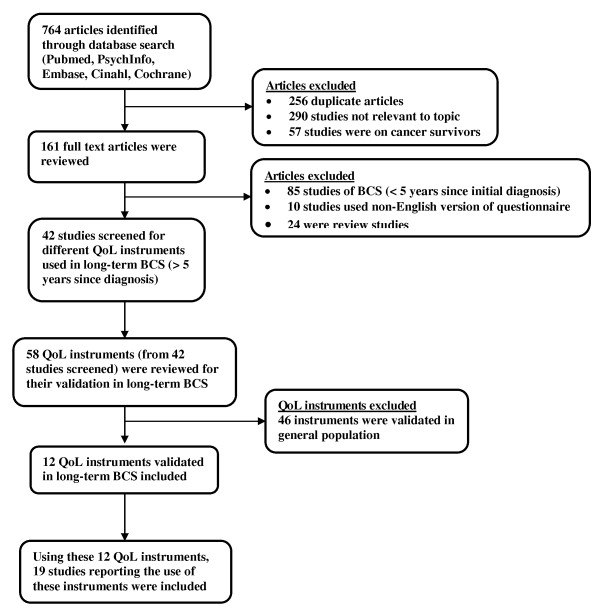
**Schematic presentation of methodology used and selection criteria**. Search and selection criteria conducted in accordance with PRISMA (Preferred Reporting Items for Systematic Reviews and Meta-Analyses) statement criteria [12].

Following the Preferred Reporting Items for Systematic Reviews and Meta-Analyses (PRISMA) guidelines [12], a systematic literature search was conducted among peer-reviewed journals from January 1990 to October 2010 by the first author in electronic databases such as Pubmed, PsychInfo, Embase, Cinahl, and Cochrane review (Figure 2). For the purpose of the review, long-term breast cancer survivor was defined as an individual who had survived five or more years since the diagnosis of cancer [6]. The search strategy included the following keywords or their combinations: *quality of life, health-related quality of life, measures, scales, questionnaires, breast cancer, breast carcinoma, breast cancer survivors, long-term breast cancer survivors, post-treatment, post-chemotherapy, post-radiation therapy*, and *post-surgery*. The search was conducted to identify studies reporting the use of QoL instruments in the evaluation of breast cancer and its treatment in long-term BCS. The QoL instruments used in these studies were also identified. These QoL instruments were then reviewed for their validation in long-term BCS. The literature search process is illustrated in Figure 2.

### Inclusion and exclusion criteria

Instruments were included in the review if they were used in at least one long-term BCS study and their description and psychometric properties were reported in BCS population with a varying number of survivor years. All included instruments were identified as patient-reported outcome questionnaires measuring one or more aspects of QoL (physical, psychological, social, and spiritual). The instruments that measured patient satisfaction or patient preference were excluded. Also, the instruments without any description of their development or validation were excluded from the review.

In addition to the inclusion of instruments, the review also included studies in long-term BCS that used these validated instruments. The search was limited to studies in English language and the use of the English version of the QoL instrument. The ACS's definition of long-term survival (> 5 years since diagnosis) was used [6]; studies of BCS of less than five years after initial diagnosis were excluded. Some studies reporting QoL outcomes for survivors with a varying number of years after diagnosis were included only if a measure of time post-diagnosis/treatment was included, the mean post-diagnosis years (≥ 5 years) was reported, and the results were presented separately for long-term survivors. Randomized clinical studies focusing on breast cancer treatments and review studies evaluating QoL in BCS were excluded. Also excluded from the review were conference abstracts, dissertations, commentaries, editorials, or summary reports on QoL. The inclusion of articles was limited to BCS; studies on cancer survivors in general were excluded.

### Data extraction

There are generally three types of QoL instruments: generic, disease-specific, and condition-specific. The generic instruments are designed to measure the complete spectrum of disease in various populations and are useful in comparing QoL changes across different diseases [13]. The disease-specific instruments assist in the measurement of domains of QoL specific to a particular disease. The condition-specific instruments measure change in specific conditions related to a disease, such as fatigue [13]. In addition to identifying the type of QoL instrument, information on items, domains, domain description, scaling and scoring, and administration of each QoL instruments was extracted.

The information on psychometric properties of the instrument (reliability, validity, and responsiveness) was also extracted. The QoL instruments are generally tested for two types of reliability: internal consistency reliability and test-retest reliability. The internal consistency reliability is measured as Cronbach's alpha (α) whereas the test-retest reliability is estimated as Pearson product-moment correlation coefficient (r). The validity refers to the degree to which an instrument measures the concept it is intended to measure. The responsiveness is the ability to detect change in health status over time [13].

For the studies reporting the use of QoL instruments, the following information was collected: sample size, socio-demographic variables (age, ethnicity, employment status, education, and number of years since diagnosis), medical variables (type of treatment and tumor stage at diagnosis), inclusion/exclusion criteria used in the selection of population for their studies, QoL instruments used, administration of QoL instrument, and survivor's QoL.

## Results

### Validated QoL measures in long-term BCS

A total of 12 QoL instruments (10 disease-specific and two condition-specific) were identified and included in the review [14-27]. The disease-specific instruments were further categorized into cancer-specific and breast cancer-specific instruments. The cancer-specific instruments included Functional Assessment of Chronic Illness Therapy-Spiritual Well Being Scale (FACIT-SP), Quality of Life-Cancer Survivor (QOL-CS), Ferrans and Powers's Quality of Life Index-Cancer Version (QLI-CV), Quality of Life in Adult Cancer Survivors Scale (QLACS), Cancer Rehabilitation Evaluation System Cancer-Short Form (CARES-SF), European Organization for Research and Treatment of Cancer (EORTC QLQ-C30), Functional Assessment of Cancer Therapy-General (FACT-G), and Body Image and Relationships Scale (BIRS). The breast cancer-specific instruments mostly used along with cancer-specific instruments included European Organization for Research and Treatment of Cancer-Breast Module (EORTC QLQ-BR23) and Functional Assessment of Cancer Therapy-Breast (FACT-B). Fatigue Symptom Inventory (FSI) and Multidimensional Fatigue Symptom Inventory (MFSI) were the two condition-specific instruments validated in long-term BCS and were included in the review.

### Description of the instruments and their psychometric properties

The instruments varied widely in number of items and domains, mode of administration, scaling and scoring, and psychometric properties. The different measures used in BCS and their complete description such as items, domains, scaling, scoring, and means of administration is provided in Table 1. The number of items and domains in the instruments ranged from 12-83 and 2-12, respectively. According to the QoL framework proposed by Ferrell *et al*., three of the identified disease-specific instruments (QOL-CS, QLACS, and QLI-CV) evaluated all four domains (physical, psychological, social, and spiritual) of QoL and included items consistent with survivor's concerns [20,24,25]. Other disease-specific instruments (EORTC QLQ-C30, EORTC QLQ-BR23, FACT-G, FACT-B, CARES-SF, and BIRS) included both physical and psychosocial domains [14-19,21,22], while FACIT-SP assessed only the spiritual aspect of QoL [23]. There are only two condition-specific instruments (FSI and MFSI) validated in BCS and both measure fatigue [26,27]. In terms of administration, most of the instruments such as EORTC QLQ-C30, EORTC QLQ-BR23, FACIT-SP, QOL-CS, QLI-CV, and MFSI could be self-administered or administered by an interviewer. The administration time for some instruments, such as EORTC QLQ-C30, FACT-G, FACT-B, and MFSI, was reported to range from 5 to 15 minutes.

**Table 1:** Description of quality of life instruments in long-term breast cancer survivors.

Instruments	Domains (Items)	Domain description	Ferrell's QoL domains *				Scaling & scoring/Administration
				
			**Ph**.	**Ps**.	**So**.	**Sp**.	
**Disease-specific measures**							

Body Image and Relationships Scale (BIRS) [14]	3 (32)	Strength and health; Social barriers; Appearance and sexuality	✓	✓	✓		5-point Likert scale; 1 (Strongly disagree) to 5 (strongly agree). Total and subscale score = summing all item scores. Higher score indicates greater impairment. Self-administered

Cancer Rehabilitation Evaluation System Cancer-Short Form (CARES-SF) [15]	6 (59)	Global CARES-SF; Physical; Psychosocial; Medical interaction; Marital relationship; Sexual concerns	✓	✓	✓		5-point scale ranging from 0 ("not at all"; no problem) to 4 ("very much"; severe). Higher scores represent poorer QoL. Self-administered

European Organization for Research and Treatment of Cancer (EORTC QLQ-C30) [16-18]	10 (30)	Functional domains: Physical; Role; Emotional; Cognitive; Social; Global QoL Symptom domains: Fatigue; Nausea/vomiting; Pain Single-item domain: Dyspnea, appetite loss, sleep disturbance, constipation, diarrhoea	✓	✓	✓		Physical and role function, dichotomous (Yes/No); Global QoL, 7-point scale; Other items, 4-point Likert scale ranging from 1 (not at all) to 4 (very much). Scale scores = mean of item scores, rescaled to 0 to 100, with higher function subscale scores indicating less dysfunction and higher symptom subscale scores indicating more dysfunction. Self-administered, interviewer-administered. Time to administer: 15 min.

European Organization for Research and Treatment of Cancer-Breast Module (EORTC QLQ-BR23) [19]	8 (23)	Body image; Sexual functioning; Arm symptoms; Breast symptoms; Sexual enjoyment; Systemic therapy side-effects; Future perspective; Upset by hair loss	✓				4-point scale ranging from 1 (not at all) to 4 (very much). Scale scores = mean of item scores, rescaled to 0 to 100. Self-administered, interviewer-administered

Ferrans and Powers's Quality of Life Index-Cancer Version (QLI-CV) [20]	2 (70)	Satisfaction with various domains of life (Part 1): Health and functioning; Socioeconomic; Psychological/spiritual; Family Importance of the same domains to the subject (Part 2): Health and functioning; Socioeconomic; Psychological/spiritual; Family	✓	✓	✓	✓	6-point Likert-type scale ranging from 1 (very dissatisfied) to 6 (very satisfied) for PART 1, and 1 (very unimportant) to 6 (very important) for PART 2. Overall & Subscale scores range from 0 to 30. Higher scores indicate better QoL. Self-administered, interviewer-administered

Functional Assessment of Cancer Therapy-Breast (FACT-B) [21]	7 (44)	Emotional well-being; Functional well-being; Physical well-being; Social/family well-being; Relationship with doctor; Breast cancer subscale; Additional concerns	✓	✓	✓		5-point Likert scale; 0 (not at all) to 4 (very much). Total FACT-B score has a range of 0 to 144. Self-administered. Time to administer: 10 min.

Functional Assessment of Cancer Therapy-General (FACT-G) [22]	5 (28)	Emotional well-being; Functional well-being; Physical well-being; Social/family well-being; Relationship with doctor	✓	✓	✓		5-point Likert scale; 0 (not at all) to 4 (very much). Item scores are summed to form overall and subscale scores. Higher scores indicate less dysfunction. Self-administered. Time to administer: 5 min.

Functional Assessment of Chronic Illness Therapy-Spiritual Well Being Scale (FACIT-SP) [23]	2 (12)	Faith; Purpose				✓	5-point scale ranging from 0 (not at all) to 4 (very much). Higher scores indicate stronger spiritual beliefs. Self-administered, interviewer-administered

Quality of life-Cancer Survivor (QOL-CS) [24]	4 (41)	Physical well-being; Psychological well-being; Social well-being; Spiritual well-being	✓	✓	✓	✓	Visual analog scale ranging from 0 (worst) to 1 (best). Average of Scores (Overall & Subscale scores range from 0 to 1). Higher scores indicate better QoL. Self-administered, interviewer-administered

Quality of Life in Adult Cancer Survivors Scale (QLACS) [25]	12 (47)	Generic domains: Physical pain; Negative feelings; Positive feelings; Cognitive problems; Sexual problems; Social avoidance; Fatigue Cancer-specific domains: Distress about family; Distress about recurrence; Appearance concerns; Benefits of cancer; Financial problems resulting from cancer	✓	✓	✓	✓	Each item score range from 1 (Never) to 7 (Always). Scores for each domain are the sum (after appropriate reverse scoring) of the individual item scores. Domain scores range from 4-28 points. Higher scores represent poorer QoL. Self-administered

**Condition-specific measures**							

Fatigue Symptom Inventory (FSI) [26]	3 (13)	Intensity of fatigue; Interference of fatigue; Fatigue duration	✓				Visual analog scale, 0 to 10. Intensity of fatigue (0 = not at all fatigued and 10 = extreme fatigue); Interference of fatigue (0 = no interference and 10 = extreme interference); Fatigue duration (0 = none of the day and 10 = the entire day). Scores range from 0-96. Higher scores indicating greater impact of fatigue. Self-administered

Multidimensional Fatigue Symptom Inventory (MFSI) [27]	5 (83)	Global fatigue; Somatic symptoms; Affective symptoms; Behavioral symptoms; Cognitive symptoms	✓				5-point scale ranging from 0 (not at all) to 4 (extremely). Self-administered, interviewer-administered Time to administer: 5-10 min.

* Ph. = Physical; Ps. = Psychological; So. = Social; Sp. = Spiritual

The psychometric properties of the instruments used in BCS are presented in Table 2. All the QoL instruments reviewed in the study reported an overall internal consistency reliability (α) in the range of 0.70-0.98. Additionally, QLACS reported α for specific domains: generic (α = 0.95) and cancer-specific (α = 0.98). QOL-CS, FACT-G, and FACT-B also reported test-retest reliability which ranged from 0.85 to 0.92. The instruments were also tested for one or more validity measures with criterion-related and construct validity being the most commonly used validity measures. Besides reliability and validity, responsiveness was reported for QLACS, EORTC QLQ-BR23, FACT-B, and MFSI. An overall psychometric review of the instruments reveal that QLACS has a high internal consistency reliability, validity, and responsiveness compared to other instruments in BCS. The generic measures have not been validated in breast cancer population and were not included in this review.

**Table 2:** Psychometric properties of quality of life instruments in long-term breast cancer survivors.

Instruments	Reliability	Validity	Responsiveness
**Disease-specific measures**			

Body Image and Relationships Scale (BIRS) [14]	Internal consistency = 0.94	Convergent and divergent	Not Reported

Cancer Rehabilitation Evaluation System Cancer-Short Form (CARES-SF) [15]	Internal consistency (for domains, 0.85-0.61)	Concurrent	Not Reported

European Organization for Research and Treatment of Cancer (EORTC QLQ-C30) [16-18]	Internal consistency > 0.70	Content, concurrent, discriminant	Not Reported

European Organization for Research and Treatment of Cancer-Breast Module (EORTC QLQ-BR23) [19]	Internal consistency: American sample (0.70-0.91); Dutch sample (0.57-0.89); Spanish sample (0.46-0.94)	Content, construct, criterion-related	Dutch &Spanish sample showed responsiveness in side effects & body image; no responsiveness tested in American sample.

Ferrans and Powers's Quality of Life Index-Cancer Version (QLI-CV) [20]	Internal consistency = 0.95	Concurrent (criterion-related, r = 0.80), construct.	Not Reported

Functional Assessment of Cancer Therapy-Breast (FACT-B) [21]	Internal consistency = 0.90 Test-retest = 0.85	Content, construct, concurrent (r = 0.87), divergent, known group	Sensitivity to 2-month changes found for FACT-B global, FACT-G global, physical well-being, functional well-being, breast cancer subscale.

Functional Assessment of Cancer Therapy-General (FACT-G) [22]	Internal consistency = 0.89 Test-retest = 0.92	Content, construct, divergent, known group.	Not Reported

Functional Assessment of Chronic Illness Therapy-Spiritual Well Being Scale (FACIT-SP) [23]	Internal consistency = 0.81-0.88	Discriminant, convergent	Not Reported

Quality of Life-Cancer Survivors tool (QOL-CS) [24]	Internal consistency = 0.93 Test-retest = 0.89	Content, concurrent (r = 0.78), predictive, construct, discriminate	Not Reported

Quality of Life in Adult Cancer Survivors Scale (QLACS) [25]	Internal consistency (generic = 0.95, cancer-specific = 0.98)	Concurrent, retrospective	Change in health status

**Condition- specific measures**			

Fatigue Symptom Inventory (FSI) [26]	Internal consistency > 0.70	Convergent, divergent, construct	Not Reported

Multidimensional Fatigue Symptom Inventory (MFSI) [27]	Internal consistency = 0.87-0.92	Convergent and divergent	Significant differences in expected direction were found between cancer patients and non-cancer patients.

### Review of studies using validated QoL measures in long-term BCS

Based on the literature search methodology, 19 studies were identified that reported the use of QoL instruments in long-term BCS [28-46]. Table 3 provides a summary of the studies including information regarding sample size, survivor's age, number of years of post-diagnosis, inclusion/exclusion criteria of the study, survivorship, QoL instruments used, administration of QoL instrument, and survivor's QoL.

**Table 3:** Quality of life studies in long-term breast cancer survivors.

Study	Study objective	Patient population	Inclusion/exclusion criteria	Instruments used/ Administration	Results	Ferrell's QoL domains *
Dow *et al*. (1996) [28]	Evaluation of QoL in long-term BCS.	N = 294; mean post-cancer survivorship = 68.5 months; mean age = 50.9 years.	Not reported	QOL-CS, FACT-G Self-administered	Concerns included psychological/family distress, fear of recurrence, uncertainty, fatigue, chest pain, sleep problems, and sexuality.	Ph. Ps. So. Sp.

Weitzner *et al*. (1997) [29]	Comparison of mood and QoL of BCS with those observed in low-risk breast cancer screening patients.	Long-term stage I-III BCS (N = 60); mean age = 53.8 years. Low-risk breast cancer screening patients (N = 93); mean age = 45.3 years. Mean post-cancer survivorship ≥ 5 years.	Inclusion (BCS): disease-free ≥ 5 years; stage I, II, or III; age < 70 years. Inclusion (comparison group): no personal/family history of breast cancer. Exclusion (both groups): DSM-III-R psychiatric diagnosis; brain carcinoma; using steroids/narcotic analgesics.	QLI-CV; other instruments (BDI, STAI) Self-administered	Stage III breast cancer resulted in significantly poorer functioning compared to other groups.	Ps. So.

Ashing-Giwa *et al*. (1998) [30]	Evaluation of QoL of long- term BCS and to examine the role of ethnicity.	African-American: N = 117; mean post-cancer survivorship = 6.5 years; White respondents: N = 161); mean post-cancer survivorship = 7.4 years.	Inclusion: breast carcinoma diagnosis between 1989 and 1990; previously participated in a study of first-degree relatives of BCS by Bastani *et al*.	CARES-SF; other instruments (SF-36, Ladder of Life Scale, Life Distress Scale). Self-administered	Overall, BCS reported favorable health-related QoL. Differences in QoL outcomes were attributable to socioeconomic and life-burden factors and not to ethnicity.	Ph. So.

Holzner *et al*. (2001) [31]	Evaluation of effect of time elapsed since initial diagnosis on QoL.	N = 87; mean post-cancer survivorship = 5.1 years; mean current age = 53.9 years.	Inclusion: Relapse-free patients. Exclusion: presence of other severe diseases.	EORTC QLQ-C30, EORTC QLQ-BR23, FACT-B. Self-administered	Emotional, social, and sexual functioning areas showed reduced QoL after initial treatment (1-2 years) and > 5 years survival.	Ps. So.

Beaulac *et al*. (2002) [32]	Evaluation of effect of surgical treatment related lymphedema on QoL.	Women with lymphedema, (N = 42), without lymphedema (N = 109); mean post-cancer survivorship = 5.0 years; mean age = 62.4 years.	Inclusion: mastectomy or breast-conserving surgery with radiation; level I, II axillary lymph node dissection. Exclusion: other breast surgery; chemotherapy; rheumatologic condition.	FACT-B. Self-administered	Women with lymphedema reported lower breast, functional, and physical wellbeing, irrespective of type of surgery.	Ph. Ps. So.

Cimprich *et al*. (2002) [33]	Evaluate relationship between life-stage variables on QoL in BCS.	Diagnosis age: young (< 45 years, N = 42), middle (45-65 years, N = 35), old (> 65 years, N = 28); mean post-cancer survivorship = 11.5 years.	Inclusion: at least 5 years past the diagnosis; no recurrent disease or other cancer diagnosis.	QOL-CS Self-administered	Long-term BCS diagnosed at an older age had worse QoL in physical domain and women diagnosed at a younger age had worse QoL in social domain.	Ph. Ps. So.

Kornblith *et al*. (2003) [34]	Assessing the long-term impact of breast carcinoma in BCS.	Phase III randomized trial CALGB 7581] group; N = 153; mean post-cancer survivorship = 18 years; age (range) = 41-87 years.	Inclusion: no evidence of breast carcinoma; completion of all cancer treatment at least1 year interview; no major psychiatric/cognitive deficit.	EORTC QLQ-C30; other instruments (BSI, LES, OARS, PCL-C). nterviewer-administered	Persistent psychological effects were observed in BCS long after treatment completion.	Ph. Ps. So.

Sammarco *et al*. (2003) [35]	Evaluation of relation among social support, uncertainty, and QoL in older BCS.	Older women (> 50 years); N = 103; mean post-cancer survivorship = 5.0 years; mean age = 68 years.	Inclusion: > 50 years; able to read and respond in English.	QLI-CV; other instruments (SSQ, MUIS-C). Self-administered	There was significant association between perceived social support and QoL. Uncertainty resulted in poorer QoL.	Ph. Ps. So. Sp.

Casso *et al*. (2004) [36]	Assessing QoL of long-term BCS diagnosed at age of 40-49 years.	N = 216; mean post-cancer survivorship = 7.3 years; age (range) = 45-60 years.	Inclusion: women with an initial diagnosis of ductal carcinoma *in situ *(DCIS) or invasive breast cancer; diagnosis age of 40-49 years.	CARES-SF; other instruments (SF-36, CES-D). Self-administered	Long-term QoL affected by surgery/chemotherapy/hormonal therapy. Negative impact of breast related symptoms/pain on QoL.	Ph. So.

Ahles *et al*. (2005) [37]	Comparison of local therapy and standard-dose systemic chemotherapy on BCS's QoL.	Women treated with standard-dose systemic chemotherapy (N = 141, mean age = 57 years) or local therapy (N = 294, mean age = 65.8 years); mean post-cancer survivorship = 10.0 years.	Inclusion: > 5 years after diagnosis; currently disease free; currently receiving no cancer treatments.	QOL-CS. Interviewer-administered	Survivors treated with systemic chemotherapy exhibited lower overall QoL compared with survivors treated with local therapy only.	Ph. Ps. So.

Burckhardt *et al*. (2005) [38]	Evaluation of effect of chronic pain on health status and overall QoL resulting from surgical treatment.	Women with regional pain: N = 11; mean post-cancer survivorship = 5.9 years; mean age = 58.7 years and with widespread pain: N = 12; mean post-cancer survivorship = 5.4 years; mean age = 56.8 years.	Inclusion: post-mastectomy ≥ 6 months/≥ 3 months post-radiation/cytotoxic chemotherapy; cancer-free; simple mastectomy/lumpectomy/modified radical mastectomy. Exclusion: breast surgery for cosmetic reasons or prophylactic mastectomy; arthritis.	FACT-B, QOLS; other instruments (BPI, MPQ-SF, FIQ, SF-36). Self-administered	Women who experienced widespread pain after breast cancer surgery had significantly more severity of pain and lower physical health status than those with regional pain.	Ph. Ps. So.

Helgeson *et al*. (2005) [39]	Examining the impact of breast cancer on long-term QoL.	Survivors, N = 267, mean age = 54.4 years. Controls, N = 187, mean age = 53.2 years; mean post-cancer survivorship = 5.5 years.	Inclusion: Women with Stage I, II, or III breast cancer who underwent surgery followed by adjuvant chemotherapy between 1993 and 1996.	FACIT-SP, MFSI; other instruments (SF-36, PANAS, BSI, DAS, IES). Interviewer-administered	Survivors reported more difficulties with physical functioning and more physical symptoms.	Ph. So.

Carver *et al*. (2006) [40]	Assessing the effect of medical, demographic, and personal variables on BCS's QoL.	N = 163; mean post-cancer survivorship = 10.0 years; mean age at diagnosis = 54.2 years.	Inclusion: Women who participated in the past projects (1988-1995, 1994-1996).	QLACS; other instruments (LOT, ISEL). Self-administered	Initial chemotherapy and higher stage predicted more financial problems and worry about appearance. More distress and social avoidance in Hispanic BCS.	Ph. Ps. So.

Dirksen *et al*. (2007) [41]	Evaluation of efficacy of cognitive behavioral therapy on fatigue, mood, and QoL in BCS.	N = 86; mean post-cancer survivorship, CBT group = 85.3 months & control = 63.8 months; mean age = 58 years.	Inclusion: stage I, II or III, ≥ 3 months post-treatment; disease-free; sleep problem ≥ 3 months. Exclusion: cognitive impairment or other sleep disorders (restless leg syndrome).	FACT-B; other instruments (STAI, CES-D, POMS). Self-administered	Women receiving cognitive behavioral therapy for insomnia had significant improvements in fatigue, trait anxiety, depression and QOL.	Ph. Ps. So.

Perkins *et al*. (2007) [42]	Evaluation of individual differences in well-being in older BCS.	N = 127; mean post-cancer survivorship = 5.1 years; mean age = 78.2 years.	Inclusion: BCS survivors with a current age of 70 or older.	FACIT-SP, FSI; other instruments (SF-36, LOT-R). Self-administered	Higher age predicted increased depression. Poorer health status was associated with poorer well-being.	Ph. Ps. So. Sp.

Leak *et al*. (2008) [43]	Examining relation among symptom distress, spirituality, and QoL of African- American BCS.	N = 30; mean post-cancer survivorship = 5.6 years; mean age 55.5 years.	Inclusion: speak and read English; had diagnosis of breast cancer; treatment completion (Jan 1, 1980 - June 1, 2004). Exclusion: recurrent breast cancer/another cancer in the past 12 months.	QLI-CV; other instruments (SDS, SPS). Interviewer -administered	Sleep disturbance, fatigue, and pain were the most commonly reported symptoms in African- American BCS.	Ph. Ps. So. Sp.

Sammarco *et al*. (2008) [44]	Examining relation among perceived social support and uncertainty on Hispanic BCS's QoL.	N = 89; mean post-cancer survivorship = 5.0 years; age (range) = 30-86 years.	Inclusion: at least one year after treatment.	QLI-CV; other instruments (SSQ, MUIS-C). Self-administered	Perceived social support and uncertainty play a pivotal role in managing or maintaining QoL in Hispanic BCS.	Ph. Ps. So. Sp.

Skrzypulec *et al*. (2008) [45]	Evaluate problems related to total and partial mastectomy affecting QoL.	Treatment with total mastectomy (N = 403, mean age = 57.8 years); partial mastectomy (N = 91, mean age = 47.3 years); post-cancer survivorship = 6-10 years.	Inclusion: total mastectomy due to breast cancer (research group); partial mastectomy (control group). Exclusion: using drugs impeding sexual function.	EORTC QLQ-BR23; other instruments (IES, LSI, HADS). Self-administered	The level of depression and anxiety in women after mastectomy results in worse bio-psychosocial functioning.	Ph. So.

Speck *et al*. (2010) [46]	Evaluation of impact of Physical Activity and Lymphedema (PAL) trial on perceptions of body image in BCS.	BCS with lymphedema (N = 112); without lymphedema (N = 122). Post-cancer survivorship > 5 years.	Inclusion: unilateral non-metastatic breast cancer; BMI < 50 kg/m^2^; cancer-free; no medical conditions limiting participation in exercise program; no weight lifting in year prior to study entry, not currently pregnant/lactating.	BIRS Self-administered	Twice-weekly strength training positively impacted self-perceptions of appearance, health, physical strength, sexuality, relationships, and social functioning.	Ph. So.

* Ph. = Physical; Ps. = Psychological; So. = Social; Sp. = Spiritual

Other instruments: instruments validated in general population and used in the included studies.

BDI = Beck Depression Inventory; BIRS = Body Image and Relationships Scale; BPI = Brief Pain Inventory; BSI = Brief Symptom Inventory; CALGB = Cancer and Leukemia Group B; CARES-SF = Cancer Rehabilitation Evaluation System Cancer-Short Form; CBT = Cognitive Behavioral Therapy; CES-D = Center for Epidemiologic Studies Depression Scale; DAS = Dyadic Adjustment Scale; EORTC QLQ-C30 = European Organization for Research and Treatment of Cancer; EORTC QLQ-BR23 = European Organization for Research and Treatment of Cancer-Breast Module; FACT-B = Functional Assessment of Cancer Therapy-Breast; FACT-G = Functional Assessment of Cancer Therapy-General; FACIT-SP = Functional Assessment of Chronic Illness Therapy-Spiritual Well Bring Scale; FIQ = Fibromyalgia Impact Questionnaire; FSI = Fatigue Symptom Inventory; HADS = Hospital Anxiety and Depression Scale; IES = Impact of Events Scale; LES = Life Experience Survey; LSI = Life Satisfaction Index; ISEL = Interpersonal Support Evaluation List; LOT = Life Orientation Test; LOT-R = Life Orientation Test Revised; MFSI = Multidimensional Fatigue Symptom Inventory; MPQ-SF = Short Form McGill Pain Questionnaire; MUIS-C = Mishel Uncertainty in Illness Scale-Community; OARS = Older American Services and Resources Questionnaire; PANAS = Positive and Negative Affect Scale; PCL-C = Posttraumatic Stress Disorder Checklist-Civilian; POMS = Profile of Mood States; QLACS = Quality of Life in Adult Cancer Survivor Scale; QLI-CV = Quality of Life Index-Cancer Version; QOL-CS = Quality of Life-Cancer Survivor SF-36 = Medical Outcomes Study 36-item Short Form Health Survey; SDS = Symptom Distress Scale; SPS = Spiritual Perspective Scale; SSQ = Social Support Questionnaire; STAI = State-Trait Anxiety Inventory

Most of the studies utilized two or more QoL instruments to assess different aspects of QoL in long-term BCS. Cancer-specific QoL instruments designed to measure physical and psychosocial domains were used by six studies [28,30,31,34,36,46], while the cancer-specific QoL instruments (QOL-CS, QLACS, QLI-CV) that measured all four aspects of QoL were used by nine studies [28,29,33,35,37,38,40,43,44]. The breast cancer-specific measures designed to evaluate physical and psychosocial aspects specific to breast cancer were used by five studies [31,32,38,41,45]. Two studies have used FACIT-SP, an instrument for measuring spiritual aspect of QoL [39,42]. Condition-specific instruments were used by two studies to measure fatigue in BCS [39,42].

The results from the 19 studies are summarized into six categories, although some studies met the criteria for more than one category. These include overall QoL and effect of different demographic and medical variables (life stage, old age, ethnicity, breast cancer treatment, and non-pharmacological intervention) on QoL.

#### Overall QoL

Five studies assessed the effect of breast cancer and its treatment on overall QoL among long-term BCS [28,29,34,39,40]. The results of the study by Dow *et al. *[28] indicated both positive and negative long-term effects. The positive outcomes included hopefulness and presence of positive change after treatment. The major concerns included fatigue, aches and pains, sleep problems, psychological distress from cancer diagnosis and treatment, fear of recurrence, family distress, sexuality, family burden, and uncertainty which had negative impact on overall QoL. Helgeson *et al. *[39] indicated difficulties in physical functioning in disease-free BCS. Additionally, the survivors with recurrence had poor QoL compared to the survivors without recurrence. Weitzner *et al. *[29] assessed overall QoL as well as relationship between mood and QoL. They compared long-term BCS with women in breast cancer screening group. The results showed that BCS had a high incidence of symptoms related to depression and trait anxiety, resulting in lower QoL. The patients with advanced stage of cancer had poorer QoL. Kornblith *et al. *[34] reported that majority of the BCS presented a significant recovery from their cancer diagnosis and treatment, but some survivors reported cancer-related emotional and medical problems due to lymphedema and numbness in hands and feet that interfered with their QoL. Carver *et al. *[40] investigated the effect of medical, demographic, and psychosocial variables on different aspects of QoL. The study showed that medical variables such as stage of disease and treatment intensity were strongly associated with financial problems and worries about appearance. The psychosocial variables, such as earlier trait optimism, confidence about remaining cancer free, and perceptions of available social support predicted variations in psychosocial aspects of QoL. The demographic variables including older age at cancer diagnosis, more time elapsed since diagnosis, being non-Hispanic white, being more educated, and having employment predicted lower psychosocial distress.

#### Life stage and QoL

Three studies examined the relationship between life stage variables (survivor's age at diagnosis and number of years of post-diagnosis survivorship) and QoL [31,33,36]. Cimprich *et al. *[33] categorized their sample into three groups based on their age at diagnosis: younger age (27-44 years), middle-aged (45-65 years), and older age (> 65 years). The younger-age at diagnosis group showed poorer outcomes in social aspect, with major concerns being changes in self-concept and appearance. The group of patients who had received diagnosis at an older age had the worst QoL in relation to physical well-being, with major problems being fatigue, pains, sleep changes, and constipation. There were differences in the psychological aspect also. The concerns of older-age at diagnosis group included feeling less useful in life and uncertainty about the future. The younger-age group at diagnosis showed psychological distress related to family distress, diagnosis, and treatment. Interestingly, the QoL improved with an increase in the number of post-diagnosis years. Casso *et al. *[36] used a sample of BCS who were diagnosed between the ages of 40-49 years. Their findings suggested a varied response to QoL depending on the type of breast cancer treatment received. Women who received adjuvant systemic therapy had poorer QoL outcomes in physical, psychosocial, and sexual aspects compared to women who did not receive adjuvant systemic therapy. Similarly, women who had mastectomy reported more physical concerns compared to women who had breast conserving therapy. Moreover, the presence of breast-related symptoms such as pain, swelling, and numbness resulted in poor QoL. These concerns, however, were not affected by the number of years post-diagnosis. The study by Holzner *et al. *[31] focused on the effect of time elapsed since initial diagnosis on QoL. The reduced QoL was observed not only in patients in the first two years after initial treatment but also in patients with a survival time of more than 5 years. The authors observed a "rebound effect" (a recurring reduction of QoL after initial improvement) in their study, particularly in the psychosocial aspect of QoL. It was suggested that the long-term BCS's concerns arose with the necessity for regular aftercare visits, chronic physical long-term effects of cancer treatment (arm swelling or scar pain), and fear of possible recurrence which makes the survivors continually aware of the illness they have survived. A similar pattern was observed for the aspect of role functioning, where patients reported the difficulties in occupational tasks, organizing leisure time activities, and pursuing their hobbies.

#### Age of breast cancer survivor and QoL

Two studies examined the effect of different age groups on QoL [35,42]. Sammarco *et al. *[35] assessed QoL in BCS older than 50 years of age. A significant association was observed between perceived social support and the physical aspects of QoL. The findings suggested that the unpredictable nature of breast cancer and its treatment coupled with the presence of other diseases or functional disabilities of aging resulted in poorer QoL, especially in the health/functioning, socioeconomic, and psychological/spiritual domains. Moreover, fear of recurrence, fear of death and suffering, and concerns regarding payment for the healthcare costs affected QoL among older survivors. The increased uncertainty in illness negatively impacted their QoL. Perkin *et al. *[42] evaluated QoL in survivors aged 70 years or older and reported a significant association between health status variables of physical functioning, life satisfaction, depression, and general health perceptions.

#### Ethnicity and QoL

Four studies evaluated the relationship between long-term effects of breast cancer on QoL and ethnicity [30,40,43,44]. Sammarco *et al. *[44] assessed the overall QoL among Hispanic BCS. The findings showed that perceived social support and uncertainty were important in providing improved QoL in this population. The Hispanic survivors experienced a higher degree of uncertainty and were not aware of the available social support resources which affected their QoL. Their major concerns included family distress, socioeconomic problems, and problems with health and functioning. Another study by Carver *et al. *[40] in the Hispanic population, observed that Hispanic women reported elevations in several kinds of problems: negative feelings, social avoidance, distress about the family's future, and distress about the possibility of recurrence compared to all other ethnic groups. Leak *et al. *[43] examined the overall QoL among African-American BCS. Sleep disturbance, pain, and fatigue were the most commonly reported symptoms in this population. The overall high QoL scores were attributed to the relatively high level of spirituality, which helped them cope with cancer. Ashing-Giwa *et al. *[30] also compared the overall QoL among African-American BCS with Caucasian BCS and reported an improved health status and fairly good overall QoL in both the groups. The differences in QoL between the groups were not attributable to ethnicity; instead they resulted from differences in the socioeconomic and life-burden factors.

#### Breast cancer treatment and QoL

Four studies evaluated the effect of breast cancer treatment on different aspects of QoL or overall QoL [32,37,38,45]. Beaulac *et al. *[32] examined the effect of lymphedema on QoL resulting from surgical treatment. The authors observed that lymphedema resulted in decreased range of arm motion and women with lymphedema reported lower breast, functional, and physical wellbeing. However, the lymphedema related problems were not affected by the type of surgical treatment (mastectomy or breast conserving surgery). Additionally, Burckhardt *et al. *[38] focused on BCS with regional pain and widespread pain resulting from surgical treatment. Most of the patients experienced chronic pain immediately after surgery or in the early post surgical treatment period which had lingering effects. However, the chronic pain also originated later in the post-surgical period. Skrzypulec *et al. *[45] examined the effect of surgical treatment focusing on total and partial mastectomy. Mastectomy resulted in physical limitations and interfered with the psychological and social functioning in these women, which in turn adversely affected everyday QoL and lowered the long-term QoL. Ahles *et al. *[37] focused on long-term BCS who were treated with either standard dose systemic chemotherapy or local therapy (treating a lesion or tumor). There was a significant negative impact on the social and physical aspects of QoL in survivors treated with chemotherapy compared to women treated with local therapy. The major physical problems were fatigue, pain, overall physical health, and menstrual/fertility problems, while the social concerns included interference with activities at home, employment, and financial burden.

#### Non-pharmacological intervention and QoL

Two studies evaluated the effect of non-pharmacological intervention on QoL among long-term BCS [41,46]. Dirksen *et al. *[41] reported short-term positive changes in fatigue, trait anxiety, depression, and QoL following cognitive behavioral therapy (CBT) for insomnia in BCS. Perkins *et al. *[46] showed that Physical Activity and Lymphedema trial (PAL) had beneficial effects on self-perceptions of appearance, health, physical strength, sexuality, relationships, and social functioning, as measured by the Body Image and Relationships Scale.

## Discussion

The transition of a patient with breast cancer from treatment phase to survivorship phase is an important aspect of cancer continuum. The number of BCS and the number of survivorship years will increase with further advancements in breast cancer treatments. However, these therapies have persistent side-effects and toxicity which have been shown to negatively impact a survivor's QoL [8]. Thus, there is a pressing need to understand and monitor the prolonged effects of breast cancer and its treatment so as to capture the concerns of the survivors and convey the information to clinical decision-makers who can use it to create patient-centered solutions.

To date, QoL assessments in cancer survivors and in BCS in particular, have employed several valid instruments that assess different QoL dimensions. As indicated by Ferrell and colleagues, consideration of psychological, social, physical, and spiritual aspects of QoL is essential in understanding the long-term impact of breast cancer diagnosis and treatments. Furthermore, few instruments are available that specifically evaluate all the different aspects of QoL in BCS. Three cancer-specific instruments, QLACS, QLI-CV, and QOL-CS evaluate all four domains of QoL and include questions specifically relevant to BCS. The EORTC and FACT instruments are important for measuring cancer-specific concerns, but they lack some survivor-specific concerns such as fear of recurrence, compromise with physical problems, and psychosocial issues. These cancer-specific instruments have shown similar results related to survivor's QoL when used in different studies focusing on BCS. For instance, presence of physical problems such as fatigue, pain, and insomnia as well psychosocial problems such as anxiety, depression, and concerns about appearance were consistently measured by these instruments.

Another important criterion for the selection of QoL instruments is to ensure that the instruments have good psychometric properties. Almost all the instruments in BCS have reported good psychometric properties such as reliability and validity. However, most of the instruments have not been tested for responsiveness, an important property for assessing the change in QoL over time. The concerns related to different aspects of QoL change as a survivor transitions to another year of survivorship. The change may be positive or negative based on the survivor's perceptions, expectations, and overall health. It is, thus, important for the instruments to capture the changes in patient outcomes that vary with time. Additionally, none of the generic measures have been validated in a breast cancer population, creating a need for validation of these measures for evaluating changes in self-reported general health status of patients with cancer as they transition from treatment phase to survivorship. The generic instruments provide insight into the complete spectrum of disease and are useful not only in comparing QoL changes across different populations (for example, patients undergoing breast cancer treatment, patients with metastatic breast cancer, and breast cancer survivors) but also across different diseases (for example, comparing BCS with ovarian or cervical cancer survivors). There are certain domains of QoL that are more prominent in BCS. For instance, fatigue, pain, insomnia, depression, anxiety, and fear of recurrence are persistent concerns that affect survivors and these conditions could affect their activities of daily living, coping skills, and social and role functions. The review identified only two condition-specific instruments (FSI and MFSI, measuring fatigue) that have been validated in BCS. However, these instruments do not fully assess the concerns of the survivors resulting in a need for validating the existing condition-specific instruments. Also, there are some disease-specific instruments such as Breast Cancer Prevention Trial (BCPT) Symptoms Scale [47], Impact of Cancer Scale (IOCv2) [48], and Long-term Quality of Life (LTQL) [49] which have been validated in BCS population; however, we were unable to locate studies utilizing these instruments in BCS that met our inclusion criteria. Overall, QLACS demonstrated good reliability, validity, and responsiveness in long-term BCS compared to other instruments that were reviewed.

Another goal of this review was to evaluate the studies that assessed QoL in long-term BCS using validated instruments. These studies assessed inter-connectedness and the important aspects of Ferrell's QoL domains. Most of the studies showed that these domains are inter-related. The physical concerns such as sexuality and menopausal symptoms impact both psychological and social aspects of QoL. The social concerns including changes in self-concept as well as physical concerns negatively impact psychological well-being. The spiritual well-being helps in improving physical, psychological, and social well-being by providing strength to cope with negative effects of breast cancer and its treatment.

The studies also evaluated specific concerns of BCS related to each domain. First, the physical domain of QoL was most commonly evaluated by the studies, with most frequent issues being fatigue, sleep problems, and pain. Additionally, lymphedema (swelling of arms) was reported only in women treated with breast cancer surgery. These physical problems are typical for breast cancer treatment and have long-lasting effects; becoming even more problematic with increase in age. Second, the psychological well-being was also most frequently evaluated, with major symptoms being emotional distress and depression resulting from concerns such as fear of recurrence, uncertainty, family distress, financial burden, and worries about appearance. Higher level of psychological distress was reported in Hispanic women. Third, the social well-being was affected by absence of sufficient social support. This was a major concern in women 50 years and older as well as in Hispanic women. Fourth, spiritual well-being is also an important aspect of QoL, but is least often reported by the studies. Spiritual well-being was evaluated by two studies, one reported post-treatment positive change in life, while other study reported good overall QoL in African-American BCS resulting from higher levels of spirituality.

In addition, these studies assessed specific concerns of BCS related to different demographic and medical variables. The studies reported an overall improvement in QoL in BCS, but the findings varied considerably based on the demographic and medical variables. For instance, the BCS diagnosed at a younger age experience problems with social well-being compared to the BCS diagnosed at an older age experiencing problems with physical well-being. The Hispanic women experience a higher degree of uncertainty compared to women of other ethnic groups. Moreover, the survivor's concerns vary with type of breast cancer treatment. Presence of lymphedema is common for BCS treated with mastectomy and concerns such as fatigue and pain are common for BCS treated with chemotherapy. Thus, the knowledge of variability in survivor's concerns is important for clinicians and decision-makers to help guide proper follow-up care after completion of breast cancer treatment.

Most of the studies have focused on QoL during breast cancer diagnosis and treatment, but there is a gap in the literature for studies focusing on long-term BCS. There is a need for studies comparing and evaluating the effects of different breast cancer therapies (surgical treatment, systemic therapies-chemotherapy, hormone therapy, radiation therapy, newer targeted therapies with monoclonal antibodies, and adjuvant endocrine therapy) in long-term BCS. More studies are required comparing QoL in BCS diagnosed at different stages of breast cancer as the extent of treatment and its effects might vary at different stages of cancer. There is a need for studies assessing impact of co-morbid conditions in older (> 60 years) BCS. Moreover, the studies analyzing the problems resulting from cancer recurrence are important. The studies reported in this review are cross-sectional studies, thus do not provide change in QoL over time in BCS. These studies provide varied results in different groups of BCS, which makes it difficult to generalize these results to the general population of BCS. Most of the studies included in this review have used more than one instrument, in which one or two instruments have been validated in the breast cancer population; other instruments used have only been validated in general population. The spiritual outcomes are the least reported, and consideration of the spiritual domain in future studies is important.

## Conclusions

This review highlights the significant and persistent effects of breast cancer and its treatment on long-term BCS and the inter-connectedness of the psychological, social, physical, and spiritual aspects of QoL in BCS. Thus, clinicians and decision-makers need to understand the complexity of problems in long-term BCS to help guide proper follow-up care after the completion of breast cancer treatment. This review also provides useful insight into the unique concerns and needs of BCS, which can help researchers and clinicians select the most appropriate instruments to assess the changes in QoL. The use of validated instruments will not only provide valid data but also help improve the quality of care in long-term BCS.

## Abbreviations

ACS: American Cancer Society; BCS: Breast Cancer Survivors; BDI: Beck Depression Inventory; BIRS: Body Image and Relationships Scale; BPI: Brief Pain Inventory; BSI: Brief Symptom Inventory; CALGB: Cancer and Leukemia Group B; CARES-SF: Cancer Rehabilitation Evaluation System Cancer-Short Form; CBT: Cognitive Behavioral Therapy; CES-D: Center for Epidemiologic Studies Depression Scale; DAS: Dyadic Adjustment Scale; EORTC QLQ-BR23: European Organization for Research and Treatment of Cancer-Breast Module; EORTC QLQ-C30: European Organization for Research and Treatment of Cancer; FACIT-SP: Functional Assessment of Chronic Illness Therapy-Spiritual Well Being Scale; FACT-B: Functional Assessment of Cancer Therapy-Breast; FACT-G: Functional Assessment of Cancer Therapy-General; FIQ: Fibromyalgia Impact Questionnaire; FSI: Fatigue Symptom Inventory; HADS: Hospital Anxiety and Depression Scale; IOC: Impact of Cancer Scale; IES: Impact of Events Scale; LES: Life Experience Survey; LSI: Life Satisfaction Index; ISEL: Interpersonal Support Evaluation List; LOT: Life Orientation Test; LOT-R: Life Orientation Test-Revised; LTQL: Long-term Quality of Life; MFSI: Multidimensional Fatigue Inventory; MPQ-SF: Short Form McGill Pain Questionnaire; MUIS-C: Mishel Uncertainty in Illness Scale Community; OARS: Older American Services and Resources Questionnaire; PAL: Physical Activity and Lymphedema Trial; PANAS: Positive and Negative Affect Scale; PCL-C: Posttraumatic Stress Disorder Checklist-Civilian; POMS: Profile of Mood States; QLACS: Quality of Life in Adult Cancer Survivors Scale; QLI-CV: Quality of Life Index-Cancer Version; QOL: Quality of Life; QOL-CS: Quality of Life-Cancer Survivors; QOLS: Quality of Life Scale; SF-36: Medical Outcomes Study 36-item Short Form Health Survey; SDS: Symptom Distress Scale; SPS: Spiritual Perspective Scale; SSQ: Social Support Questionnaire; STAI: State-Trait Anxiety Inventory.

## Competing interests

The authors declare that they have no competing interests.

## Authors' contributions

IC contributed to the conception, design, collection and analysis of studies, and organized the complete draft. KK conducted a critical review of the manuscript for important intellectual content and provided finishing touch to the manuscript. Both authors read and approved the final manuscript.
